# Novel Biomarkers of Gastric Adenocarcinoma: Current Research and Future Perspectives

**DOI:** 10.3390/cancers13225660

**Published:** 2021-11-12

**Authors:** Nadja Niclauss, Ines Gütgemann, Jonas Dohmen, Jörg C. Kalff, Philipp Lingohr

**Affiliations:** 1 Department of General, Visceral, Vascular and Thoracic Surgery, University Hospital Bonn, 53127 Bonn, Germany; jonas.dohmen@ukbonn.de (J.D.); kalff@uni-bonn.de (J.C.K.); philipp.lingohr@ukbonn.de (P.L.); 2 Department of Pathology, University Hospital Bonn, 53127 Bonn, Germany; ines.guetgemann@ukb.uni-bonn.de

**Keywords:** gastric cancer, advanced gastric cancer, biomarker, targeted therapy

## Abstract

**Simple Summary:**

Gastric cancer is characterized by poor survival rates despite surgery and chemotherapy. Current research focuses on biomarkers to improve diagnosis and prognosis, and to enable targeted treatment strategies. The aim of our review was to give an overview over the wide range of novel biomarkers in gastric cancer. These biomarkers are targets of a specific treatment, such as antibodies against human epidermal growth factor receptor 2. Other promising biomarkers for targeted therapies that have shown relevance in clinical trials are vascular endothelial growth factor, programmed cell death protein 1, and Claudin 18.2. There is a vast number of biomarkers based on DNA, RNA, and protein expression, as well as detection of circulating tumor cells and the immune tumor microenvironment.

**Abstract:**

Overall survival of gastric cancer remains low, as patients are often diagnosed with advanced stage disease. In this review, we give an overview of current research on biomarkers in gastric cancer and their implementation in treatment strategies. The HER2-targeting trastuzumab is the first molecular targeted agent approved for gastric cancer treatment. Other promising biomarkers for targeted therapies that have shown relevance in clinical trials are VEGF and Claudin 18.2. Expression of MET has been shown to be a negative prognostic factor in gastric cancer. Targeting the PD-1/PD-L1 pathway with immune checkpoint inhibitors has proven efficacy in advanced gastric cancer. Recent technology advances allow the detection of circulating tumor cells that may be used as diagnostic and prognostic indicators and for therapy monitoring in gastric cancer patients. Prognostic molecular subtypes of gastric cancer have been identified using genomic data. In addition, transcriptome profiling has allowed a comprehensive characterization of the immune and stromal microenvironment in gastric cancer and development of novel risk scores. These prognostic and predictive markers highlight the rapidly evolving field of research in gastric cancer, promising improved treatment stratification and identification of molecular targets for individualized treatment in gastric cancer.

## 1. Introduction

Gastric cancer (GC), based on GLOBOCAN 2020 data [1], is the fifth most common cancer and the fourth most common cause of cancer-related death in the world. Gastric adenocarcinomas account for 5.6% of all new cancer cases and 7.7% of all cancer deaths worldwide [1]. H. pylori infection is the strongest known risk factor for gastric cancer [2]; another pathogen associated with gastric cancer is the Epstein–Barr virus [3]. The incidence of gastric cancer has steadily declined worldwide over the past 50 years, due to prevention and treatment of H. pylori infection and changing of food preservation and diet [4]. Surgery associated with chemotherapy still offers the best chance for curative therapy. Due to earlier detection of GC and achievements in chemotherapy and targeted therapy, mortality has decreased in recent decades. Still, the overall survival of gastric cancer remains low, with a reported 5-year survival rate of 32% in all stages combined, and of only 6% in metastatic disease [5]. This is mostly due to the fact that gastric cancer is usually diagnosed in an advanced and unresectable stage. If the cancer is diagnosed and treated before it has spread outside the stomach, the 5-year survival rate is 70% [5]. Therefore, most current new strategies aim to detect gastric cancer at an early stage, or to treat gastric cancer at an advanced stage. Biomarkers are playing a crucial role in these strategies. Cancer biomarkers can be soluble molecules derived from tumor cells, or can be soluble or cell-bound molecules that are expressed by nontumorous cells. Genetic, epigenetic, proteomic, glycomic, and imaging biomarkers can be used for cancer diagnosis, prognosis, and epidemiology [6]. In recent decades, multiple novel biomarkers have been identified in GC, and biomedical sciences and technology have developed at a rapid pace. The Cancer Genome Atlas (TCGA) project has identified four major genomic subtypes of GC: Epstein–Barr Virus (EBV)-infected tumors, tumors with microsatellite instability (MSI), genomically stable tumors, and chromosomally unstable tumors, which might provide a guide to targeted agents [7].

This review intends to give an overview of the literature on current and newly identified biomarkers and their roles in targeted therapies in gastric cancer (i.e., their function as predictive markers). PubMed was searched for articles using the terms ‘biomarker’ and ‘gastric cancer’ on 28 March 2021. We analyzed English articles from the last 10 years including clinical trials and randomized controlled trials. We obtained 295 articles. We excluded 150 articles that did not mention biomarkers in gastric or esophagogastric cancer or did not differentiate results between either gastric or esophagogastric and other carcinomas. The resulting 145 articles were analyzed. Another 49 articles were cited that were found relevant to this article. Articles treating gastro-esophageal junction (GEJ) adenocarcinoma were included in this review. Furthermore, clinical implementation of these biomarkers for early diagnosis, prognosis, and prediction of drug efficacy is discussed.

## 2. Treatment-Related Biomarkers—Molecular Targeted Therapy

### 2.1. Human Epidermal Growth Factor Receptor 2

Human epidermal growth factor receptor 2 (HER2), also called ERBB2, is a receptor tyrosine-protein kinase. It is an important biomarker and key driver of tumorigenesis in GC [8]. HER2-positive tumors show *HER2* gene amplification that is generally, although not always, associated with protein overexpression, leading to tumorigenesis [9]. *HER2* acts as an oncogene, mainly because high-level amplification of the gene induces protein overexpression in the cellular membrane and subsequent acquisition of advantageous properties for a malignant cell [10]. *HER2* gene amplification can be detected by fluorescence in situ hybridization (ISH), whereas overexpression of HER2 protein is commonly assessed by immunohistochemistry (IHC). Concordance between positive gene amplification and protein overexpression has been observed in 96% of GC, whereby positive *HER2* amplification was defined as a *HER2*/chromosome 17 centromere (CEP17) ratio ≥ 2.0 [11].

HER2-positivity rates by IHC in GC range between 10.9 and 27% [11,12,13,14,15,16]. HER2-positivity rates are higher in papillary and tubular adenocarcinoma compared to poorly differentiated adenocarcinoma or signet-ring cell carcinoma [13]. For clinical use, it has been proposed to test the HER2 status in all adenocarcinoma of the stomach and carcinomas of the GEJ by IHC first. In inconclusive cases, *HER2* amplification status needs to be assessed with ISH [17].

HER2-targeted therapy has dramatically improved outcomes for HER2-positive gastric cancer. Trastuzumab is a monoclonal antibody targeting the HER2-receptor, causing downregulation of HER2. The Trastuzumab for Gastric Cancer (ToGA) trial showed improved overall survival (OS) of patients treated with trastuzumab in combination with cisplatin and a fluoropyrimidine compared to chemotherapy alone in patients with HER2-overexpressing advanced gastric or GEJ cancer (13.8 vs. 11.1 months, *p* = 0.005) [8]. A subgroup analysis of Japanese patients confirmed the benefit of adding trastuzumab to chemotherapy [18]. Trastuzumab in combination with chemotherapy is the standard of care when treating HER2-positive metastatic gastric and GEJ cancers. Furthermore, it is the first molecular targeted agent approved as standard treatment in gastric cancer.

A retrospective analysis compared OS in advanced GC patients according to HER2 status and exposure to trastuzumab. It showed longer OS of HER2-positive patients treated with trastuzumab than HER2-negative patients (24.7 vs. 13.9 months, *p* = 0.03), with trastuzumab having a significant impact on OS. Interestingly, HER2-positive patients not treated with trastuzumab showed similar OS as HER-negative patients (13.5 vs. 13.9 months, *p* = 0.91). The authors concluded that trastuzumab improved prognosis of HER2-positive beyond that of HER2-negative AGC patients, but HER2 status itself without targeted therapy might have a small impact on survival in advanced GC [19].

Li et al. analyzed whether clinicopathological factors were predictive for progression-free survival (PFS) of patients with trastuzumab-based first-line therapy. They found only liver metastasis and poor performance status to be independently associated with worse PFS [20].

Antibody–drug conjugates with trastuzumab that have been developed and tested are listed in Table 1 [21,22,23,24].

The use of trastuzumab in combination with paclitaxel in patients with tumor progression after first-line chemotherapy with or without trastuzumab did not show benefit on OS, PFS, and overall response rate (ORR) compared to chemotherapy alone in patients with HER2-positive advanced gastric or GEJ cancer [25,26].

Several phase 2 trials have tested different chemotherapies in combination with trastuzumab as first line therapy in advanced or metastatic gastric or GEJ cancer. Studies that combined trastuzumab and different chemotherapies and treatment outcomes are summarized in Table 1 [27,28,29,30,31,32,33,34,35,36,37,38,39].

Pertuzumab, another monoclonal antibody against HER2, was added to first-line trastuzumab and chemotherapy in the JACOB trial, and did not show a significant survival benefit [40,41].

While the irreversible pan-HER inhibitor dacomitinib showed only a limited response [42], poziotinib, another irreversible pan-HER inhibitor targeting EGFR, HER2, and HER4, showed an objective response rate of about 20% [43] (Table 1). The dual blockade with a pan-HER inhibitor and trastuzumab might therefore be a promising strategy in trastuzumab resistance.

Several studies that analyzed the impact of HER2 status on survival and treatment response are listed in Table 2.

Taken together, the HER2 status itself without associated treatment has no direct impact on survival in patients with advanced GC, and is therefore not a prognostic factor [11,13,16]. Nevertheless, HER2 has shown to be a predictive biomarker, as high HER2 expression is associated with better treatment response [23,89]. Furthermore, HER2 expression is associated with longer survival in patients with advanced GC following HER2-directed treatment [19,44].

Treatment response to the tyrosine kinase inhibitor afatinib is associated with EGFR and HER2 coamplification [50].

### 2.2. Epidermal Growth Factor Receptor

Epidermal growth factor receptor (EGFR) overexpression is reported in 27–55% of GC, and it is associated with shortened overall survival by multivariate analysis [51].

Lapatinib is a dual tyrosine kinase inhibitor that blocks both the HER2 and epidermal growth factor receptor (EGFR) pathways. In a phase 2 trial, lapatinib was tested as first-line single therapy in metastatic GC and showed only modest activity, with a PFS of 1.9 months and an ORR of 9% (Table 1) [44]. Addition of lapatinib has not proven to be superior to conventional chemotherapy in terms of OS and PFS, neither in first- nor in second-line treatment of advanced GC [45,46,47,48]. In a phase 2 trial with lapatinib and capecitabine as first-line treatment, lapatinib induced no changes in gene expression, and no associations between single nucleotide polymorphisms and treatment outcome were found [49].

Panitumumab, a monoclonal antibody to EGFR, has shown no advantage in terms of histological response, OS, and PFS in patients with untreated advanced esophageal, gastric, or GEJ cancer when added to conventional first-line chemotherapy, compared to chemotherapy alone (Table 1) [51,52].

Similarly, nimotuzumab, also a monoclonal antibody to EGFR, in combination with irinotecan has shown no superiority in PFS compared to irinotecan alone as second-line therapy in advanced GC (Table 1). Interestingly, there was a trend toward better response rate, OS, and PFS with nimotuzumab in the subgroup with high EGFR expression levels [53].

Cetuximab, another monoclonal antibody directed against EGFR, is primarily known as a treatment in metastatic colorectal cancer. Several nonrandomized phase 2 trials without a control group have investigated cetuximab in combination with conventional chemotherapy. As a first-line treatment, overall response rates (ORR) between 45 and 65%, PFS between 5 and 9 months, and OS between 9 and 17 months have been reported (Table 1) [54,55,57]. In the randomized EXPAND trial, cetuximab was tested in combination with capecitabine and cisplatin compared to chemotherapy alone in previously untreated advanced GC without any survival benefit [56]. As a second-line therapy, addition of cetuximab to conventional chemotherapy has shown more limited treatment response (Table 1) [58].

Studies analyzing the impact of EGFR expression on survival and treatment response showed inconsistent results (Table 2). EGFR expression was associated with better OS and a higher response rate in advanced GC under EGFR-directed therapy [55,90], while other studies showed no impact of EGFR expression on survival and treatment response [57,58]. Taken together, it remains unclear if EGFR is a prognostic or predictive biomarker.

### 2.3. Vascular Endothelial Growth Factor

Vascular endothelial growth factor (VEGF) plays a role in pathogenesis and progression of GC.

Ramucirumab, a monoclonal antibody that binds to VEGF receptor-2, was tested in the RAINBOW trial in combination with paclitaxel. This was a randomized placebo-controlled and double-blind study of 665 patients with advanced gastric or GEJ cancer with disease progression on or after first-line chemotherapy. Compared to paclitaxel alone, overall survival was significantly longer with ramucirumab (Table 1) [59].

Bevacizumab, a monoclonal antibody to VEGF, was tested in the AVAGAST study in untreated patients with advanced GC. Adding bevacizumab to chemotherapy did not improve OS, but led to a longer PFS and higher ORR compared to chemotherapy alone [60]. Meulendijks et al. investigated the efficacy of bevacizumab in combination with chemotherapy in untreated advanced gastric and GEJ cancer in two phase 2 trials without a control group. In HER2-negative patients PFS was 8.3 months and OS was 12 months, while in HER2-positive patients, a combination of trastuzumab and bevacizumab led to a PFS of 10.8 months and an OS of 17.9 months (see Table 1) [61,62].

Sunitinib, a tyrosine kinase inhibitor targeting platelet-derived growth factor (PDGF) receptor and VEGFR, was tested as monotherapy in pretreated patients with advanced GC. It was associated with very limited tumor response (Table 1) [63]. Sunitinib did not improve PFS or response as an adjunct to FOLFIRI compared to FOLFIRI alone in chemotherapy-resistant GC [64].

Foretinib, another multikinase inhibitor targeting MET and VEGFR-2, lacked efficacy in metastatic GC [95].

Several studies have analyzed the impact of VEGF on survival in advanced GC (Table 2). They consistently showed a negative association between VEGF levels and survival, indicating that VEGF is a negative prognostic biomarker [58,63,64,91]. In a biomarker study from the RAINBOW trial, all analyzed biomarkers including VEGF were not predictive for ramucirumab efficacy [92].

### 2.4. Fibroblast Growth Factor Receptor

Won et al. tested the efficacy of a combined inhibition of VEGF receptors 1–3, PDGF receptor, and fibroblast growth factor receptor (FGFR) 1–3 with the tyrosine-kinase inhibitor nintedanip. In patients with metastatic esophageal or GEJ adenocarcinoma and disease progression on first-line chemotherapy, treatment with nintedanip showed no partial or complete response (Table 1) [65].

The selective FGFR 1–3 tyrosine kinase inhibitor AZD4547 was tested as a second-line therapy in patients with advanced GC in the randomized controlled SHINE study, and did not improve PFS compared to paclitaxel (Table 1) [66].

The prognostic value of FGFR was analyzed in advanced GC treated with multikinase inhibitors (Table 2). FGFR2 expression was a significant prognostic factor for PFS with pazopanib, while there was only a trend to better PFS with nintedanip [65,93].

### 2.5. Hepatocyte Growth Factor Receptor

Hepatocyte growth factor receptor (HGFR), also called c-Mesenchymal-Epithelial Transition (MET), is a tyrosine kinase receptor. MET overexpression is highly heterogenous and uncommon in GC by immunohistochemistry [96].

The MET signaling pathway plays an integral role in GC. An aberrant, overactivated MET pathway promotes disease progression, and serves as a common mechanism of resistance to *HER*-targeted therapy. Beyond anti-HER2 therapy, the MET pathway seems to be a culprit of cancer invasiveness, with MET-overexpressing tumors having poorer prognosis [97].

Rilotumumab, a monoclonal antibody to MET, was tested against placebo in combination with chemotherapy in advanced or metastatic gastric or GEJ adenocarcinoma without testing MET status. PFS was longer with rilotumumab (Table 1) [67]. Zhu and colleagues found that high rilotumumab exposure was associated with better PFS compared to low exposure and placebo among patients with MET-positive tumors (Table 1) [68]. A randomized phase 3 trial testing rilotumumab against placebo in combination with chemotherapy was stopped early due to higher mortality in the rilotumumab group (Table 1) [69]. MET positivity was defined in both trials as 25% or more of membranous staining of tumor cells in IHC.

Several other tyrosine kinase inhibitors targeting the HGF/MET pathway were studied in MET-positive gastric cancer, but no substantial benefit was proven [98]. Thus, onartuzumab was tested in a phase 3 trial against placebo in combination with chemotherapy in HER2-negative, MET-positive gastroesophageal cancer and showed no improvement in survival or response rates (Table 1) [70].

MET expression has been shown to be a prognostic factor in locally advanced gastric and GEJ cancer treated with chemotherapy and panitumumab, as it was associated with shorter PFS and OS (Table 2) [52]. Resistance to the kinase inhibitor afatinib was associated with MET amplification in advanced GC (Table 2) [50]. Hence, MET might be predictive for decreased treatment response.

### 2.6. Claudin 18.2

In normal tissue, the tight junction molecule Claudin 18.2 is only expressed on the membrane of differentiated epithelial cells of the gastric mucosa. Its expression is activated in primary and GC and GC metastases, but also in malignancies of the pancreas, esophagus, ovaries, and the lung [98]. Claudin 18.2 expression is found in 77–87% of primary GC, and in 51–80% of lymph node metastasis [98,99]. The exclusive expression of Claudin 18.2 in differentiated gastric cells, in combination with the fact that transient gastrointestinal toxicity is a frequent and manageable adverse event, makes this molecule highly attractive as a target for the development of safe and potent drugs [98].

The monoclonal antibody zolbetuximab targets Claudin 18.2. In the FAST trial, patients with advanced gastric, GEJ, or esophageal adenocarcinoma and with moderate-to-strong Claudin 18.2 expression in ≥40% of tumor cells received chemotherapy with or without zolbetuximab. Patients treated with zolbetuximab had significantly higher PFS and OS, with an even more pronounced difference in the subpopulation with very high Claudin 18.2 expression (Table 1) [71]. The ongoing SPOTLIGHT study compares the effect of zolbetuximab against placebo in combination with chemotherapy as a first-line therapy in Claudin-18.2-positive and HER-2-negative advanced gastric or GEJ cancer [100].

### 2.7. Ataxia Teleangiectasia Mutated

Ataxia telangiectasia mutated (ATM) is a key activator of DNA damage response. GC cell lines with low levels of ATM are sensitive to the poly ADP ribose polymerase (PARP) inhibitor olaparib, which prevents tumor cells from repairing DNA damage from chemotherapy. Olaparib was tested against placebo in combination with paclitaxel in patients with metastatic GC and showed improved OS in both the overall population and the population with low ATM levels, but no difference in PFS or response rates (Table 1) [72].

### 2.8. AKT

Ipatasertib is a small molecule inhibitor of AKT, a key component of the PI3K/AKT pathway. When tested in a randomized controlled trial in combination with FOLFOX6 against placebo, it did not improve PFS. No benefit was observed in biomarker-selected patients (PTEN-low, PI3K/AKT-activated tumors) [73].

### 2.9. Histone Deacetylase

Vorinostat, an inhibitor of histone deacetylase (HDAC), was investigated in combination with capecitabine and cisplatin as a first-line chemotherapy in advanced GC, and showed an ORR of 42% and a 6-month PFS rate of 44%. As in a previous phase 3 study with capecitabine and cisplatin with a 6-month PFS rate of 40%, the addition of vorinostat was not likely to enhance efficacy. A biomarker analysis using Western blotting included plasma levels of atecyl-H3, HDAC2, and p21. None of these three biomarkers correlated with PFS, but high baseline acetyl-H3 and p21 were significantly associated with worse OS [74].

### 2.10. Matrix Metalloproteinase-9

Matrix metalloproteinases are proteases involved in degradation and remodeling of the extracellular matrix and basement membranes. Matrix metalloproteinase-9 (MMP9), which is expressed heterogeneously by tumor epithelia and infiltrating inflammatory cells, has been associated with loss-of-tumor suppression activity, as well as oncogenic activity [75].

Andecaliximab, a monoclonal antibody targeting MMP9, showed encouraging results in a phase 2 trial, but failed to show improved OS in the ensuing randomized GAMMA-1 trial (Table 1) [76].

### 2.11. Immunotherapy

Programmed cell death protein 1 (PD-1) is located at the surface of immune cells, and functions as an immune checkpoint by regulating the immune response. Programmed cell death ligand 1 (PD-L1) binds to PD-1 and inhibits the immune response through inhibition of T-cell receptor-mediated lymphocyte proliferation and cytokine secretion, among other mechanisms [101]. PD-L1 expression is measured with IHC, and PD-L1 positivity is defined as a combined positivity score (CPS) ≥ 1, where CPS is the number of PD-L1-positive cells (tumor cells, lymphocytes, and macrophages) divided by the total number of viable tumor cells, multiplied by 100 [102].

In the molecular evaluation of gastric adenocarcinoma as part of the TCGA project, PD-L1/2 expression was elevated in EBV-positive tumors, suggesting that PD-L1/2 antagonists should be tested in this subgroup [7]. This was confirmed by Liu and colleagues, who found PD-L1 expression significantly associated with MSI, EBV-positive, and H. pylori status. There was a greater proportion of PD-L1 CPS ≥ 1 tumors among MSI-H versus microsatellite stable (MSS), EBV-positive versus EBV-negative, and H. pylori-positive as compared to H. pylori-negative tumors. PD-L1 CPS ≥ 1 was observed in 49.7% of EBV-negative and MSS tumors [102].

Pembrolizumab, a monoclonal antibody to PD-1, was tested in the phase 1b KEYNOTE-012 trial in patients with PD-L1-positive recurrent or metastatic gastric or GEJ adenocarcinoma, and showed an objective response rate of 22% and a rate of grade 3–4 treatment-related adverse events of 13% [77]. In the following phase 2 KEYNOTE-059 trial, pembrolizumab was tested in 259 patients with disease progression after two or more lines of chemotherapy. PD-L1 expression was assessed in tumor biopsy samples by immunohistochemistry. Tumors were considered PD-L1 positive if the combined positive score (number of PD-L1-positive cells including tumor cells, macrophages, and lymphocytes divided by the total number of tumor cells, multiplied by 100) was 1 or greater. Response to pembrolizumab treatment was observed in both PD-L1-positive and -negative tumors, but was higher in patients with PD-L1-positive compared to PD-L1-negative tumors (15.5 vs. 6.4%, *p* = 0.02). There was no difference in OS between patients with PD-L1-positive and PD-L1-negative tumors (5.8 vs. 4.9 months) (Table 1) [78].

Kim et al. observed very high ORR with pembrolizumab in patients with MSI-high (85.7%) and EBV-positive (100%) metastatic GC [79]. These results were in line with higher immunogenicity of MSI or virally induced tumors in other localizations, such as gynecologic malignancies [103].

More recently Kawazoe et al. tested pembrolizumab in combination with the oral fluorouracil derivate S-1 plus oxaliplatin as a first-line treatment in patients with PD-L1-positive and HER2-negative advanced gastric or GEJ cancer, and observed high ORR (Table 1) [80].

A recent single-arm phase 2 trial investigated the combination of pembrolizumab with a HER2-targeting antibody as a proof of concept of synergistic antitumor activity. Janjigian et al. tested trastuzumab and pembrolizumab plus conventional chemotherapy as a first-line therapy in HER2-positive metastatic gastric or GEJ cancer. The PFS at 6 months was 70% [85]. The combination of pembrolizumab and trastuzumab is currently being further tested in the ongoing KEYNOTE-811 randomized controlled trial [104].

Margetuximab, a novel anti-HER2 monoclonal antibody, was evaluated in a single-arm phase 1b-2 trial in combination with pembrolizumab in HER2-positive, PD-L1-unselected gastric or GEJ cancer on progression after chemotherapy with trastuzumab. This phase 1b/2 trial showed a considerable ORR of 18.5%. This study confirmed that combined targeting of HER2 and PD-1/PD-L1 could yield antitumor activity greater than that with either approach alone [86].

The anti-PD-L1 antibody durvalumab and the anti-CTLA-4 antibody tremelimumab were tested alone or in combination in patients with chemotherapy-refractory gastric or GEJ cancer. ORR and PFS were low and did not differ between treatment arms (Table 1) [87].

Toripalimab, a monoclonal antibody to PD-1, was given as monotherapy in a group of patients with chemo-refractory GC, and in combination with chemotherapy in a group of chemotherapy-naïve patients. With toripalimab, monotherapy ORR was 12.1%, while in combination with chemotherapy, the ORR was 66.7% (Table 1) [81].

In the ATTRACTION-2 trial, the PD-1 antibody nivolumab was tested against placebo in patients with advanced gastric or GEJ cancer refractory to two or more regimens of chemotherapy. OS was significantly longer with nivolumab compared to placebo at 2-year follow-up. The authors concluded that nivolumab might be a new treatment option for heavily pretreated patients with advanced gastric or GEJ cancer (Table 1) [82].

The CheckMate 577 trial showed that Nivolumab was efficient as an adjuvant treatment in patients with resected esophageal or GEJ cancer who had received neoadjuvant chemoradiotherapy and had residual pathological disease. Disease-free survival was 22.4 months with nivolumab compared to 11 months with placebo (*p* < 0.001) [105].

The JAVELIN Gastric 100 trial, which tested the PD-L1 antibody avelumab against chemotherapy maintenance after first-line induction chemotherapy in locally advanced or metastatic gastric or GEJ cancer, showed no superior OS with avelumab, both in an overall and PD-L1-positive population [84].

The anti-PD-1 antibody SHR-1210 was tested as a second-line treatment in advanced GC, and showed an ORR of 26.7% [83].

Studies analyzing treatment outcome dependent on PD-L1 status are shown in Table 2. ORR was higher in patients with PD-L1-positive (defined as a combined positive score of 1 or greater) compared to PD-L1-negative tumors treated with pembrolizumab [78,79]. Furthermore, PD-L1 positivity was independently associated with longer disease-free survival, regardless of PD-L1-directed treatment [94].

There was no association of PD-L1 expression with treatment outcome in advanced GC treated with toripalimab and SHR-1210 [81,83].

In conclusion, PD-L1 is a prognostic biomarker and predictive for response to pembrolizumab therapy. Based on the results of the KEYNOTE-059 trial, pembrolizumab was approved by the U.S. Food and Drug Administration (FDA) for the treatment of patients with recurrent, locally advanced, or metastatic gastric or GEJ adenocarcinoma with disease progression on or after two or more systemic therapies, and whose tumors express PD-L1 [78,106]. As mentioned above, there might be a role of combined targeting of HER2 and PD-1/PD-L1. The recently published CheckMate 577 trial showed that nivolumab is also highly efficient as an adjuvant treatment in patients at risk for recurrence, regardless of PD-L1 expression [105]. Therefore, the main role of immunotherapy may be to prevent recurrence, rather than to treat metastatic or advanced disease in the future.

Adjuvant immunotherapy with autologous cytokine-induced killer cells has been assessed in a nonrandomized study for patients after gastrectomy and subsequent chemotherapy for locally advanced GC. Compared to a control group without immunotherapy, patients treated with cytokine-induced killer cells had longer 5-year disease-free survival. For patients with intestinal-type tumors, OS and disease-free survival were significantly higher for patients with immunotherapy. Subgroup analysis of patients with diffuse or mixed-type tumors showed no survival benefit from adjuvant immunotherapy (Table 1) [88].

## 3. Diagnostic and Potential Target Biomarkers

### 3.1. DNA Methylation and Gene Expression

DNA methylation is an epigenetic mechanism leading to carcinogenesis in GC through silencing of tumor-suppressor genes and activation of oncogenes [107].

Pirini et al. found lower global DNA methylation levels in endoscopic biopsies with gastric cancer than in those with gastritis [108].

DNA methylation in the long interspersed nucleotide element-1 (*LINE-1*) is a good indicator of global DNA methylation. *LINE-1* methylation has been shown to be lower in GC tissue than in matched noncancerous gastric mucosa. In addition, analysis of *LINE-1* methylation in GC specimens of 203 patients revealed that *LINE-1* hypomethylation was significantly associated with lower OS [109].

Reprimo-like (*RPRML*) is a member of the reprimo gene family that is a group of poorly understood single-exon intronless genes, and whose loss of expression is related to increased cell proliferation and growth in gastric cancer [110]. Alarcon and colleagues observed that circulating methylated *RPRML* DNA in plasma samples significantly distinguished patients with GC from cancer-free controls, and that downregulation of *RPRML* expression was associated with poor survival in advanced GC [111].

Bcl-2 homologous antagonist killer (BAK) is a protein belonging to the BCL2 family and encoded by the *BAK1* gene. BAK promotes cell death by apoptosis. Kubo et al. showed that higher BAK protein expression in gastric cancer is associated with better chemotherapeutic histopathological response to docetaxel, and with longer survival [112].

The tumor-suppressor characteristics of cyclin-dependent kinase 10 (CDK10) have been demonstrated in nasopharyngeal carcinoma and breast cancer. You et al. investigated the expression status of CDK10 and its prognostic significance in GC. They found that CDK10 protein expression was decreased in GC, and loss of CDK10 expression correlated with advanced tumor stage and unfavorable OS. CDK10 protein expression was an independent predictor for survival [113].

The role of breast cancer 1 (*BRCA1*) gene expression by IHC in sporadic gastric cancer was investigated by Kim et al. They found reduced expression of the *BRCA1* gene associated with more advanced-stage disease, perineural invasion, and decreased disease-free survival. *BRCA1* nuclear expression < 5% was predictive for the benefit of adjuvant chemotherapy [114].

Li et al. investigated genotypic distribution of toll-like receptor 4 (*TLR4*) gene polymorphisms in Chinese patients with GC and patients with atrophic gastritis. The *TLR4-2081G/A* gene polymorphism was negatively associated with occurrence of GC, indicating an influence on GC risk [115].

Alterations of deubiquitinating enzymes have been discussed in the pathogenesis of various tumors [116]. Expression of ubiquitin-specific protease 42 (USP42) mRNA was demonstrated to be higher in GC than in nontumorous tissues, and correlated with tumor size, TNM stage, lymph node metastasis, and OS of patients with GC [116]. The expression of proteasome-associated deubiquitinating enzyme UCHL5 was analyzed in a large cohort study of 650 patients with GC undergoing surgery. Positive UCHL5 protein expression was associated with better survival in the subgroup of patients with tumors <5 cm, disease stages I-II, and age 66 years or older [117].

Two studies investigated the role of biomarkers in treatment of patients with advanced GC refractory to chemotherapy with the mechanistic target of rapamycin (mTOR) inhibitor everolimus, which inhibits the ability of mTOR to phosphorylate the ribosomal protein S6, and thereby inhibits cell-cycle progression. Both studies found high expression of phospho-S6 ribosomal protein (Ser240/4) associated with better clinical response or stable disease, and prolonged PFS [118,119].

The excision repair cross-complementing gene 1 (ERCC1) is a key enzyme in the nucleotide excision repair pathway that serves as a DNA repair mechanism. High expression of ERCC1 mRNA in endoscopic biopsies of primary GC was shown to be associated with poor prognosis, and was an independent prognostic factor for OS [120]. Several potential predictive factors of the response to 5-fluorouracil (5-FU) or prognostic factors have been reported in the metabolic pathway of 5-FU and folic acid. These include thymidylate synthase (TS) and the cytosolic enzyme dihydropyrimidine dehydrogenase (DPD). High mRNA expression of TS and DPD has been shown to predict a poor clinical outcome of treatment with 5-FU [120].

Tsuburaya et al. analyzed mRNA expression of TS, DPD, topoisomerase I, ERCC1, and thymidine phosphorylase (TP) in tumor specimens of 126 patients with advanced GC. In patients treated with S-1 plus irinotecan compared to S-1 alone, low TS, low ERCC1, and high TP mRNA levels were associated with a better prognosis [121].

Sasako et al. analyzed expression of genes involved in pyrimidine metabolism, TS, DPD, TP, and orotate phosphoribosyltransferase (OPRT). Expression of these genes was determined in patients enrolled in a trial testing S-1 as an adjuvant chemotherapy for gastric cancer. Results showed that high TS and DPD expression were associated with a better OS, whereas TP and OPRT expression were not associated with survival [122].

Hirakawa et al. analyzed protein expression of damage DNA binding protein complex subunit 2 (DDB2), which serves as an initial damage recognition factor during nucleotide excision repair, and ERCC1 by IHC in tumor tissues pretreated with the combination chemotherapy of docetaxel, cisplatin, and the oral fluorouracil derivate S-1. High expression of DDB2 and ERCC1 was more frequent in tissues of nonresponders compared to responders (p = 0.0065 and p = 0.029, respectively). The authors showed that a combination of DDB2 and ERCC1 expression could predict response or nonresponse to chemotherapy in 82.5% [123].

### 3.2. Multiple Gene Expression Signatures

With next-generation sequencing (NGS), valuable tools to study GC at the molecular level have been developed. With multiomics data from genome, transcriptome, proteome, and epigenome levels, GC can been stratified to subtypes and correlated to therapeutic outcomes [124].

Roh et al. performed a biomarker analysis on tumor samples collected from the CLASSIC trial that compared capecitabine plus oxaliplatin-based adjuvant chemotherapy to surgery alone after D2 gastrectomy [125]. The authors developed a single patient classifier (SPC) assay using a combination of gene expression of nine genes, MSI status, and EBV association to predict prognosis and responsiveness of adjuvant chemotherapy [126]. In this study, the SPC-assay score and MSI status were independent prognostic factors for disease-free survival (DFS), while EBV status was not a prognostic factor [127].

Smyth et al. analyzed 200 genes by mRNA expression from tissue samples from the MAGIC trial, in which patients had been pretreated with chemotherapy [128]. They developed a seven-gene signature assay allowing stratification of patients into two risk groups according to survival. Median OS in the high- and low-risk groups were 10.2 and 80.9 months, respectively. Risk groups and lymph node metastases (LNMs) were independent prognostic factors for OS. In patients treated with surgery only from the MAGIC trial, none of the seven genes were associated with OS. Therefore, the seven-gene signature assay might help to predict prognosis in pretreated gastric, lower esophageal, or GEJ cancer patients [129].

Sundar et al. analyzed tissue samples from patients with metastatic GC that were treated with a PD-1 inhibitor. They measured alternate promoter utilization, an epigenetic phenomenon that might be associated with immune evasion in early GC. High alternate promoter utilization was found in 33% of GC, and was associated with a lower response rate and survival. They concluded that alternate promoter utilization is a potential mechanism of resistance to immune checkpoint inhibition and a novel predictive biomarker for immunotherapy [130].

Li and colleagues performed a multiomics characterization of molecular features of GC. They performed whole-genome, whole-exome, and RNA sequencing on tumor samples from 35 GC patients before and after undergoing neoadjuvant chemotherapy. Increased MSI and mutational burden were observed in nonresponse tumors, indicating that MSI-H status may serve as a predictive marker for nonresponse to neoadjuvant chemotherapy [131]. These results were in line with previous studies indicating that MSI-H status and mismatch repair (MMR) deficiency were associated with less benefit from chemotherapy. Furthermore, a significant positive prognostic effect of MSI-H status for patients with resected gastric cancer without chemotherapy has been shown [132]. On the other hand, strong immunogenicity and widespread expression of immune-checkpoint ligands make the MSI subtype more vulnerable to the immunotherapeutic approach [133]. After analysis of individual mutated genes, only mutations of the *C10orf71* gene were associated with treatment resistance. Analysis of somatic copy number alterations revealed that amplification of the *MYC* gene, a proto-oncogene, was associated with better response to chemotherapy, while amplification of another proto-oncogene, MDM2, was associated with nonresponse [131].

Biopsies from untreated advanced gastric, GEJ, or esophageal adenocarcinoma from the REAL3 trial were assessed for *KRAS*, *BRAF*, and *PIK3CA* mutations, and *PTEN* expression. In the REAL3 trial, the therapeutic efficacy of the EGFR-antibody panitumumab was assessed in combination with chemotherapy, and showed no increase in OS [52]. Furthermore, these biomarkers were assessed in patients from the MAGIC trial. Here, peri-operative epirubicin, cisplatin, and 5-fluorouracil improved survival in patients with resectable lower esophageal, gastric, or GEJ adenocarcinoma [128]. None of the tested biomarkers predicted resistance to treatment combined with panitumumab from the REAL3 trial, or were associated with survival in patients from the MAGIC trial [134].

The NanoString gene expression system captures and counts individual mRNA transcripts by direct measurement of mRNA expression levels without enzymatic reactions or bias [135]. Das et al. used the NanoString gene expression platform to analyze 105 gastric tumors from a randomized cohort that was treated with irinotecan plus S-1 (IRI-S) versus S-1 alone [121]. Increased expression level of CD14 was significantly associated with a younger age of patients. Expression levels of the chemokines CCL5 and CXCL12 were high in the diffuse type of GC. Increased mRNA expression of *ADAMTS1*, *CCL19*, and *CXCL12* was associated with peritoneal metastasis, suggesting that these genes related to the tumor microenvironment may play a significant role in tumor progression. Elevated expression levels of the *DPYD* gene, encoding the pyrimidine catabolic enzyme in the 5-FU pathway, was associated with a younger patient age and the diffuse type of GC. Higher expression of *Wnt5A* and lower expression of *PTRF* were associated with unresectable GC and measurable lesions, respectively. *Wnt5A* downregulation was identified as a predictor of improved PFS in S-1 but not in IRI-S treatment [136].

Microarrays of biopsies from advanced GC patients before chemotherapy were used to identify biomarkers for predicting efficacy of S-1, cisplatin, and docetaxel combinatory chemotherapy. A four-gene signature was identified, including platelet-derived growth factor subunit B (*PDGFB*), polycomb group ring finger 3 (*PCGF3*), cytokine-inducible SH2-containing protein (*CISH*), and annexin A5 (*ANXA5*). *PDGFB* plays an essential role in the regulation of cell proliferation. *PCGF3* is related to the signaling pathways regulating pluripotency of stem cells. *CISH* acts as regulator of cytokine signal transduction. *ANXA5* encodes an anticoagulant protein acting as indirect inhibitor of the thromboplastin-specific complex. These four genes identified early- and nonresponders to chemotherapy with an accuracy of 100%, and hence may serve as markers for efficacy of chemotherapy [137].

### 3.3. Noncoding RNA

Different noncoding RNAs, such as long noncoding RNA (lncRNA), circular RNA (circRNA), and microRNA (miRNA), are involved in GC development [138].

The HOX transcript antisense intergenic RNA (HOTAIR), an lncRNA, has shown to play an important role during GC tumorigenesis [139]. Du and colleagues investigated genetic variations of HOTAIR and association with GC risk. They found the single nucleotide polymorphism rs4759314 to be significantly associated with increased GC risk [140].

lncRNA and miRNA have been shown to be involved in GC progression: MiRNAs function through regulation of gene expression, and their dysregulated expression has been linked to tumor development and progression [141].

MiR-34 is downregulated in GC, and has been identified as a tumor suppressor in GC [142]. Pan et al. analyzed the role of miR-34 polymorphisms in GC risk. They found that the genotype miR-34b/c rs 44938723 might have a protective effect on GC risk [143]. Mu and colleagues identified miR-193b and miR-196a as promising prognostic markers in GC [144].

MiR-26a was found to be downregulated in GC, and decreased miR-26a expression correlated with poor clinical prognosis. It was suggested that miR-26a functions as a tumor suppressor in GC development and progression, and might be a prognostic biomarker and potential therapeutic target [145].

Malhotra et al. examined 1032 microRNAs expressed in 29 cases of previously untreated advanced esophagogastric cancer. They could not identify an association between tumor epithelial microRNA expression and disease progression [146].

Ahn et al. found specific miRNA single-nucleotide polymorphisms associated with GC susceptibility and prognosis in the Korean population depending on diffuse- or intestinal-type GC [147].

A systematic review identified eight consistently upregulated miRNAs (miR-21, miR-223, miR-18a, miR-214, miR-93, miR-25, miR-106b, and miR-191) and five miRNAs that were consistently downregulated (miR-375, miR-564, miR-155, miR-148a, and miR-92) in GC. Furthermore, miR-940 and the combination of miR-21, miR-93, miR-106a, and miR-106b were identified as a diagnostic biomarker for GC, while miR-204 and miR-15a were associated with poor survival in GC [148].

Another study investigated whether circRNA is involved in pathological processes of GC. The authors found circRNA Has_circ_0000745 was downregulated in GC tissues and in plasma from patients with GC. Therefore, its expression level in plasma in combination with the CEA level might be a promising diagnostic marker for GC [149].

### 3.4. Protein Expression

The adhesion molecule cadherin-17 (CDH17) is a potential marker for GC. It has been shown to be upregulated in GC, and higher expression by IHC was associated with poorer OS [150].

Human leucocyte antigen (HLA)-G expression, which is primarily seen in the placenta and induces immune tolerance in pregnancy, has been reported in several human cancers, including GC. HLA-G may represent one of the ways tumor cells escape immunosurveillance. Immunohistochemistry in 52 GC patients showed that HLA-G-positive tumors were associated with poorer OS than HLA-G-negative tumors, and HLA-G expression was an independent predictor of OS [151].

Di Bartolomeo et al. found osteopontin overexpression by IHC to be associated with a higher risk of tumor recurrence and metastases in radically resected GC. Osteopontin overexpression was an independent prognostic factor for PFS and OS [152].

Similarly, caveolin-1 expression was associated with progression and poor prognosis in GC patients after radical gastrectomy [153].

The prognostic value of 2,3-dioxygenase in GC was analyzed by Liu and colleagues: 2,3-dioxygenase expression in GC tissue after gastrectomy was an independent prognostic factor, and high expression was associated with poor OS [154].

Expression of stromal monocarboxylate transporter 4 (MCT4), a plasma membrane transporter, and the enzyme carbonic anhydrase IX have been investigated in GC specimens of 143 patients. High stromal MCT4 expression was found in 50.3% and high carbonic anhydrase IX in 51.7% of patients. High stromal MCT and carbonic anhydrase IX expression were correlated with advanced TNM stage. High stromal MCT expression was an independent predictor of poor OS and DFS. Contrarily, carbonic anhydrase IX expression was not predictive for survival [155].

Somatostatin receptor subtype 2A (SSTR2A) and human epidermal growth factor receptor 2 (HER2) expression in GC tissues of 51 patients were analyzed by Romiti et al. They observed SSTR2A expression in 74.5% of patients with a predominance in well and moderately differentiated GC. HER2 expression, which was positive in 35% of patients, was associated with SSTR2 expression in 95% of all HER2+ cases [156].

Autocrine motility factor receptor (AMFR) is a cell-surface cytokine receptor that is involved in numerous physiological and pathological processes, including cell motility, signal transduction, and protein ubiquitination [157]. Huang et al. investigated the expression of AMFR in GC and its clinical significance. AMFR expression, which was positive in 59.8% of GC, was associated with invasion depth and LNM, and reduced OS. AMFR expression was also an independent predictor for OS and DFS. Therefore, expression of AMFR was a risk factor for poor prognosis in GC patients after resection [157].

The proteins C-X-C chemokine receptor type 4 (CXCR-4) and VEGF receptor-3 have been identified as potential new biomarkers for advanced esophagogastric carcinoma associated with lymphangiogenesis, invasion, and metastasis [158]. Thomaidis et al. analyzed the expression levels of CXCR-4 and VEGF receptor-3 in 72 patients with advanced gastric or GEJ cancer treated with fluorouracil, leucovorin, and either oxaliplatin (FLO) or cisplatin (FLP). Patients with strong expression of CXCR-4 end VEGF receptor-3 showed a trend toward better OS when treated with FPL. In contrast, patients with weak CXCR-4 and VEGF receptor-3 expression had significantly better OS when treated with FLO [158].

The protein trefoil factor 3 (TFF3) is normally not expressed in gastric mucosa, while it may be detected in cases of GC [159]. TFF3 expression in GC correlates with the occurrences of lymph node metastasis, muscularis propria invasion (≥T2), worse TNM stage, and histological type, which indicates that TFF3 may be an adverse factor in GC progression and metastasis [159].

The cytokine macrophage migration inhibitory factor (MIF) is highly expressed in various tumors, including GC, and stimulates proliferation and inhibits apoptosis in cancer cells [160]. He and colleagues observed higher MIF expression in GC compared to adjacent normal tissue, and showed that MIF expression was an independent prognostic factor for poor patient survival, as well as advanced clinical stage [160].

### 3.5. Serum Biomarkers

Serum biomarkers are usually analyzed by enzyme-linked immunosorbent assay (ELISA). However, ELISA tests have limited detection sensitivity (≥1 pM), which is insufficiently sensitive for the detection of small amounts of biomarkers in the early stages of disease or infection [161].

Angiopoietin-2 is a key driver of tumor angiogenesis. Its prognostic and predictive role was assessed retrospectively in a biomarker study of the AVAGAST trial, which had shown improved PFS but not OS with addition of bevacizumab to conventional chemotherapy in patients with advanced GC [60]. Low baseline plasma levels of angiopoietin-2 were associated with longer OS (13.7 vs. 10 months, *p* = 0.0055). While baseline angiopoietin-2 was an independent prognostic marker for OS, angiopoietin-2 levels did not predict efficacy of bevacizumab [162].

Serum pepsinogen is an established marker of chronic atrophic gastritis. Its predictive value for the development of GC was studied in the Hisayama study, which followed 2446 community-dwelling Japanese aged 40 or older for 10 years who underwent a screening examination regardless of previous history of gastritis. The authors found a serum pepsinogen I level of 59 ng/mL or less and a pepsinogen I/II ratio of 3.9 or less as most predictive for GC development, independently from H. pylori infection status and history of peptic ulcer [163]. Although various cut-off values have been suggested, pepsinogen I ≤70 ng/mL and pepsinogen I/II ratio ≤ 3 have been proposed for the prediction of chronic atrophic gastritis and GC, and have been confirmed in several meta-analyses [164].

Nagel et al. analyzed serum levels of cytokeratin-18 fragments in patients enrolled in the SUN-CASE study by comparing sunitinib or placebo as adjunct to standard chemotherapy. They found that baseline full-length cytokeratin-18 correlated with treatment failure and PFS. The cytokeratin-18 fragment M30 at day 14 was identified as an independent predictor of treatment response [165].

Serum levels of vascular adhesion protein-1 (VAP-1) were measured before treatment of operable and metastatic GC. Decreased VAP-1 levels were associated with shortened OS [166].

Chemerin is a chemokine linked to adipogenesis and chemotaxis of the innate immune system. Plasma chemerin levels analyzed in 196 GC patients before surgery were found to be higher than in 196 matched healthy controls. Plasma chemerin level was an independent predictor for OS and DFS in GC patients, with a high chemerin level associated with poor OS [167].

C-C motif chemokine ligand 22 (CCL22) is a protein secreted by dendritic cells and macrophages that interacts with cell-surface chemokine receptors. CCL22 serum levels and CCL22 expression in tumor beds were shown to be higher in GC patients than in healthy controls. Furthermore, a high CCL22 serum level before surgery was an independent risk factor for early recurrence [168].

Xu et al. found a preoperative C-reactive protein/albumin ratio of 0.131 or greater to be a predictor of early recurrence (<12 months) and of response to postoperative adjuvant chemotherapy [169]. Another study found the preoperative C-reactive protein/prealbumin ratio to be predictive of recurrence, with a higher predictive value than the C-reactive protein/albumin ratio [170].

Visfatin, also called pre-B-cell colony-enhancing factor (PBEF), is a proinflammatory cytokine secreted by adipocytes, macrophages, and inflamed endothelial tissue. High expression levels of visfatin have been found in tissues of several cancers, including GC, and were shown to be associated with poor OS [171]. Lu et al. showed higher visfatin levels in plasma of GC patients compared to healthy individuals, and found preoperative visfatin levels in GC patients to serve as an independent predictor of OS [172].

### 3.6. Peritoneal Biomarkers

Measurement of carcinoembryonic antigen (CEA) in peritoneal fluid can be used to detect cancer cells in the fluid. Fujiwara et al. determined CEA mRNA using the technique of transcriptase-reverse transcriptase concerted reaction (TRC). They observed CEA mRNA in 54% of peritoneal fluids obtained during resection of GC in 137 patients. Presence of CEA mRNA was associated with poorer OS, and it was an independent prognostic factor for survival [173].

Peritoneal lavage fluids of 140 patients with advanced GC undergoing surgery were analyzed by RT-PCR targeting the markers CEA and CK-20 mRNA. In patients with negative lavage cytology, those with both CEA and CK-20 positivity showed a poorer OS. By multivariate analysis CEA alone correlated with peritoneal recurrence, CK-20 alone correlated with OS and combination of CEA and CK-20 correlated with peritoneal recurrence and OS after surgery [174].

Xie et al. compared CEA expression levels in samples of peritoneal washing fluids during D2 resection of GC with or without complete mesogastric excision. CEA expression level after gastrectomy was lower in the group with complete mesogastric excision. In patients with low CEA expression before gastrectomy, D2 gastrectomy with complete mesogastric excision was associated with better disease-free survival [175].

### 3.7. Cell Biomarkers

Circulating tumor cells (CTCs) are metastatic cells that are released from the primary tumor into the blood stream, and are easily accessible in a liquid biopsy from peripheral blood. Several studies have shown that peripheral blood CTCs are useful to predict prognosis and monitor therapy in GC patients [176].

Pernot et al. used immunomagnetic and fluorescence imaging technology for the isolation and enumeration of CTCs in peripheral blood from patients with advanced gastric and GEJ cancer. The authors found CTC counts were significantly associated with worse survival at baseline and during treatment, with the optimal threshold at 2 CTCs [177]. This was confirmed by Sclafani and colleagues, who assessed the prevalence of CTCs in metastatic esophagogastric cancer. They found an increased response rate to chemotherapy and increased PFS and OS in patients with less than 2 CTCs compared to more than 2 CTCs detected at baseline [178].

CD44 has been identified as a GC stem cell marker. Li et al. analyzed CD44 expression on CTCs by fluorescence microscopy in peripheral blood samples from 45 GC patients before treatment. They found the presence of CD44-positive CTCs and TNM stage were independent predictors for recurrence of GC [179].

### 3.8. Tumor Microenvironment

The tumor microenvironment (TME) corresponds to the aggregation of tumor cells and neighboring nontumor cells, such as stromal and immune cells, extracellular matrix, and soluble factors. The TME has been shown to play a crucial role in tumorigenesis by activating immune cells to favor tumor growth and progression. Thus, tumor-associated macrophages and tumor-associated neutrophils can exert protumoral functions by enhancing tumor cell invasion and metastasis, angiogenesis, and extracellular matrix remodeling while inhibiting antitumoral immune surveillance [180].

Immunohistochemical analysis of 52 primary GC tissues revealed that high numbers of tumor-infiltrating Tregs and low numbers of tumor infiltrating CD8+ T cells were associated with shortened OS [151].

Tada et al. analyzed peripheral blood mononuclear cells (PBMCs) and tumor-infiltrating lymphocytes (TILs) in primary advanced GC before and after VEGFR2-targeting therapy with ramucirumab. They observed reduced effector regulatory T cells (Treg cells) and reduced PD-1 expression by CD8+ T cells in TILs compared to PBMCs after therapy. Before therapy, effector Treg cells in TILs were more frequent in patients with partial response or stable disease than those with progressive disease. Thus, effector Treg cell frequency in TILs could represent a novel biomarker for stratifying clinical responders [181].

Analysis of circulating and selected intratumoral immune cells was correlated with the Lauren classification subtype and prognosis in patients with untreated advanced GC [182]. Diffuse or mixed-type advanced GC showed lower rates of CD8+ TILs, circulating natural killer (NK) cells, and Treg cells than the intestinal type of GC. While Treg cells were not a prognostic factor, higher CD8+ TIL and NK cell numbers were associated with better OS [182].

Zeng and colleagues analyzed the TME infiltration patterns of 1524 GC patients and developed the TME score as an independent prognostic biomarker and a predictive factor for response to immune-checkpoint inhibitors. The high-TME-score GC subtype was characterized by immune activation, while the low-TME-score subtype was considered T-cell suppressive and associated with worse prognosis [183].

Li and colleagues [184] evaluated the prognostic significance of major stromal and immune cells within the TME. They identified NK cells, fibroblasts, and endothelial cells as the most robust prognostic markers, and developed a TME risk score by combining these cell types. Higher TME risk scores were consistently associated with worse survival.

Zhang and colleagues used transcriptome profiling to predict peritoneal recurrence of advanced GC. They developed an immune cell infiltration score that was an independent predictor for peritoneal recurrence [185].

Furthermore, Li et al. investigated the relationship between regulatory B (Breg) cells in peripheral blood and clinical outcome in XELOX-treated patients with advanced GC. Patients with decreased Breg frequencies after XELOX treatment had a longer PFS than those with increased Breg frequencies (7 vs. 5 months, *p* = 0.01) [186].

Platelet-to-lymphocyte ratio (PLR) has been reported to be a prognostic biomarker of GC [187]. Chen and colleagues analyzed the prognostic value of PLR in patients before neoadjuvant therapy and gastric resection. They observed better DFS and OS in patients with low PLR compared to high PLR, with a cutoff PLR value of 162 [188].

Finally, high preoperative neutrophil/lymphocyte ratio (NLR) (4 or more) in primary gastric cancer has been identified as independent risk factor for reduced survival (*p* = 0.003) [189].

## 4. Discussion

Our review gives an overview of the wide range of novel biomarkers in GC. As shown, multiple targeted therapies beyond HER2-antibodies have already been developed, and show promising results in GC.

An attempt to compare therapies targeting different biomarkers remains difficult. HER2 remains, to date, the most relevant biomarker in the targeted therapy of GC. Other promising biomarkers for targeted therapies that have shown relevance in clinical trials are VEGF, PD-1, and Claudin 18.2. Expression of MET has been shown to be a negative prognostic factor in GC.

There is a vast number of biomarkers based on DNA, RNA, and protein expression analyses, as well as detection of CTCs and more recently, the immune TME that has been proven to be prognostic factors and may be used for therapeutic stratification in the future.

Up to now, it has been difficult to predict which of these numerous biomarkers will be useful in which clinical scenario. One of the problems is the multitude of molecular markers to be assessed in a single tumor.

An efficient way to assess multiple markers is molecular profiling: Kim and colleagues performed molecular profiling on a cohort of 93 patients with advanced or metastatic GC using next-generation sequencing (NGS) and IHC. IHC comprised analysis of expression of 10 proteins, including the mismatch repair (MMR) proteins MLH1, PMS2, MSH2, and MSH6; the receptor tyrosine kinases HER2, EGFR, and MET; as well as PTEN and p53. NGS was performed with a commercially available assay that enabled detection of variants in 52 genes relevant to solid tumors. In this prospective study, one group of patients was treated with matched therapy based on NGS or IHC results. Matched therapy based on NGS included trastuzumab for *ERBB2* amplification, Akt inhibitor for *PIK3CA* mutation, and FGFR inhibitor for *FGFR2* amplification. Matched therapy based on IHC consisted of trastuzumab for ERBB2 amplification, pembrolizumab for MMR deficiency, pan-ERBB inhibitor for EGFR+, and PI3Kbeta inhibitor for PTEN loss. The nonmatched group received either ramucirumab or standard chemotherapy. The overall response rate was higher with matched compared to nonmatched therapy (55.6 vs. 13.1%, *p* = 0.001) with a trend to higher PFS with matched therapy (7.1 vs. 5.2 months, *p* = 0.7). The authors concluded that, as the matched group experienced significantly better responses and survival, their pilot study justified the need for further umbrella trials in GC [190]. Umbrella trials are prospective clinical trials that test multiple targeted interventions for a single disease based on predictive biomarkers or other predictive patient risk factors [191].

Future studies should include gene panels and not only gene classifiers to cover a large number of genes and potential targets for future therapy.

Genomic profiling often presents practical challenges due to tissue availability [190]. There is certainly great potential for circulating biomarkers from liquid biopsies due to their availability. Beyond CTC and circulating tumor DNA, other circulating biomarkers such as RNA, proteins, and metabolites are still in early phases of development, and need to be explored further before broad clinical use as screening or monitoring markers [192].

From an economical point of view, molecular profiling might at this point be reserved for patients with GC resistant to chemotherapy or with metastatic disease. As techniques of molecular profiling are further improved, however, they will become more readily available and less expensive in the future.

## 5. Conclusions

In conclusion and from a clinical point of view, biopsies from patients with locally advanced or metastatic GC should be tested for HER2 overexpression, as trastuzumab is indicated in HER2-positive tumors in combination with palliative chemotherapy. Before instauration of palliative chemotherapy, tumors should also be tested for Claudin 18.2 overexpression, as targeted therapy to Claudin 18.2 has proven efficacy. In case of resistance to first-line chemotherapy, VEGF is a promising target. Tumors refractory to two or more regimens of chemotherapy should be tested for PD-L1 expression, as immune checkpoint inhibitors have proven efficacy. MSI-high tumors have shown to be especially responsive to immunotherapy. Testing tumors for MET expression might be predictive for decreased treatment response.

## Figures and Tables

**Table 1:** Targeted therapies and treatment outcomes.

Target	Study	Design	Patient Number	Treatment Aim	Outcome
HER2	Bang, Y.J. et al. [8]	RCT (phase 3)	594	Trastuzumab + CT vs. CT alone in HER2(+) AGC (ToGA trial)	OS: 13.8 vs. 11.1 months (*p* = 0.005) PFS: 6.7 vs. 5.5 months (*p* = 0.0002) ORR: 47 vs. 35% (*p* = 0.002)
	Sawaki, A. et al. [18]	RCT (phase 3)	101	Trastuzumab + CT vs. CT alone in HER2(+) AGC (subgroup analysis of ToGA trial)	OS: 15.9 vs. 17.7 months PFS: 6.2 vs. 5.6 months ORR: 64.4 vs. 58.5%
	Shitara, K. et al. [19]	Retrospective case series	364	Trastuzumab + CT in HER2(+) vs. CT in HER2(−) AGC (1), CT in HER2(+) vs. HER2(−) AGC (2)	OS: 24.7 vs. 13.9 months (*p* = 0.03) (1) OS: 13.5 vs. 13.9 months (*p* = 0.91) (2)
	Li, Q. et al. [20]	Prospective observational study	107	Trastuzumab as first-line treatment in HER2(+) AGC	OS: 16 months PFS: 7.7 months ORR: 58.9%
	Shitara, K. et al. [21]	RCT (phase 2)	187	Trastuzumab deruxtecan vs. CT in previously treated HER2(+) AGC	OS: 12.5 vs. 8.4 months (*p* = 0.01) ORR: 51 vs. 14% (*p* < 0.001)
	Thuss-Patience, P.C. et al. [22]	RCT (phase 2/3)	182	Trastuzumab emtansine vs. Taxane as second-line therapy in HER2(+) AGC (GATSBY study)	OS: 7.9 vs. 8.6 months (*p* = 0.86)
	Shah, M.A. et al. [23]	RCT (phase 2/3)	182	Biomarker analysis of the GATSBY study: Trastuzumab emtansine vs. Taxane as second-line therapy in HER2(+) AGC	Subgroup with high HER2 expression in IHC: OS: 9.5 vs. 8.3 months
	Shitara, K. et al. [24]	RCT (phase 2/3)	82	Trastuzumab emtansine vs. taxane as second-line therapy in HER2(+) AGC (subgroup analysis of GATSBY study)	OS: 11.8 vs. 10 months
	Horita, Y. et al. [25]	Phase 2	28	Paclitaxel + trastuzumab in previously treated HER2(+) AGC	OS: 9.6 months PFS: 4.6 months ORR: 21.4%
	Makiyama, A. et al. [26]	RCT (phase 2)	91	Paclitaxel + trastuzumab vs. Paclitaxel as first-line therapy of HER2(+) AGC	OS: 10 months (*p* = 0.20) PFS: 3.7 vs. 3.2 months (*p* = 0.33) ORR: 33 vs. 32% (*p* = 1.00)
	Ryu, M.H. et al. [27]	Phase 2	55	Trastuzumab + capecitabine + oxaliplatin in HER2(+) AGC	OS: 21 months PFS: 9.8 months ORR: 67%
	Gong, J. et al. [28]	Phase 2	51	Trastuzumab + oxaliplatin + capecitabine as first-line therapy in HER2(+) AGC	OS: 19.5 months PFS: 9.2 months ORR: 66.7%
	Rivera, F. et la [29]	Phase 2	41	Xelox + trastuzumab as first-line therapy of HER2(+) AGC	OS: 13.8 months PFS: 7.1 months ORR: 46.7%
	Roviello, G. et al. [30]	Phase 2	15	DOF (docetaxel, oxaliplatin, 5-FU) + trastuzumab in HER2(+) AGC	OS: 19.4 months PFS: 9.2 months ORR: 60%
	Mondaca, S. et al. [31]	Phase 2	26	mDCF (docetaxel, cisplatin and 5-FU) + trastuzumab as first-line therapy in HER2(+) metastatic GC	OS: 24.9 months PFS: 13 months ORR: 65%
	Kagawa, S. et al. [32]	Phase 2	23	Trastuzumab + docetaxel as first-line therapy in HER2(+) AGC	OS: 17.5 months PFS: 6.7 months ORR: 39.1%
	Takahari, D. et al. [33]	Phase 2	75	Trastuzumab + S-1 + oxaliplatin in HER2(+) AGC	OS: 18.1 months PFS: 8.8 months ORR: 70.7%
	Yuki, S. et al. [34]	Phase 2	42	Trastuzumab + S-1 + oxaliplatin as treatment of HER2(+) advanced or recurrent GC	OS: 27.6 months PFS: 7.0 months ORR: 82.1%
	Kataoka, H. et al. [35]	Phase 2	22	Trastuzumab + S-1 + cisplatin in HER2(+) AGC	OS: 15.3 months PFS: 7.5 months ORR: 41.2%
	Miura, Y. et al. [36]	Phase 2	44	Trastuzumab + S-1 + cisplatin in HER2(+) AGC	OS: 16.5 months PFS: 5.9 months ORR: 61%
	Endo, S. et al. [37]	Prospective observational study	15	Trastuzumab + cisplatin + S-1 in HER2(+) AGC	OS: 14.4 months
	Kimura, Y. et al. [38]	Phase 2	51	Trastuzumab + S-1 in patients 65 years or older with HER2(+) AGC	OS: 15.8 months PFS: 5.1 months ORR: 40.8%
	Shah, M.A. et al. [39]	RCT (Phase 3b)	248	Standard-of-care vs. higher-dose trastuzumab + CT as first-line therapy in HER2(+) metastatic GC (HELOISE trial)	OS: 12.5 vs. 10.6 months (*p* = 0.2401)
	Tabernero, J. et al. [40]	RCT (phase 3)	780	Pertuzumab + trastuzumab + CT vs. placebo + trastuzumab + CT as first-line therapy of HER2(+) AGC (JACOB trial)	OS: 17.5 vs. 14.2 months (*p* = 0.057)
	Liu, T. et al. [41]	RCT (phase 3)	163	Pertuzumab + trastuzumab + CT vs. placebo + trastuzumab + CT as first-line therapy of HER2(+) metastatic GC (subgroup analysis of JACOB trial)	OS: 18.7 vs. 16.1 months PFS: 10.5 vs. 8.6 months ORR: 68.9 vs. 55.7%
	Oh, D.Y. et al. [42]	Phase 2	27	Dacomitinib in previously treated HER2(+) AGC	OS: 7.1 months PFS: 2.1 months ORR: 7.4%
	Kim, T.Y. et al. [43]	Phase 2	32	Poziotinib + trastuzumab + paclitaxel as second-line therapy in HER2(+) AGC	OS: 29.5 weeks PFS: 13 weeks ORR: 21.9%
EGFR HER2	Iqbal, S. et al. [44]	Phase 2	47	Lapatinib as first-line therapy in advanced or metastatic GC	OS: 4.8 months PFS: 1.9 months ORR: 9%
	Satoh, T. et al. [45]	RCT (phase 3)	261	Lapatinib + paclitaxel vs. paclitaxel alone as second-line therapy in HER2(+) AGC	OS: 11 vs. 8.9 months (*p* = 0.10) PFS: 5.4 vs. 4.4 months (*p* = 0.24) ORR: 27 vs. 9% (*p* < 0.001)
	Lorenzen, S. et al. [46]	RCT (phase 2)	37	Lapatinib + capecitabine vs. lapatinib alone in HER2(+) AGC	ORR: 11.1% (LAP + CAP) (study closed for futility)
	Hecht, J.R. et al. [47]	RCT (phase 3)	545	Lapatinib + capecitabine/oxaliplatin vs. placebo + capecitabine/oxaliplatin in HER2(+) AGC	OS: 12.2 vs. 10.5 months (NS) PFS: 6.0 vs. 5.4 months (*p* = 0.038) ORR: 53 vs. 39% (*p* = 0.003)
	Moehler, M. et al. [48]	RCT (phase 2)	29	Lapatinib + ECF/ECX vs. placebo + ECF/ECX as first-line therapy in metastatic GC patients EGFR(+) and/or HER2(+)	OS: 13.8 vs. 10.1 months (NS) PFS: 8 vs. 5.9 months (NS) ORR: 42.9 vs. 21.4%
	LaBonte, M.J. et al. [49]	Phase 2	68	Lapatinib as first-line therapy in AGC independent of HER2 status	OS: 6.3 months PFS: 3.3 months ORR: 17.9%
	Sanchez-Vega, F. et al. [50]	Prospective observational study	20	Afatinib in trastuzumab-resistant HER2(+) metastatic GC	OS: 7 months PFS: 2 months ORR: 25%
EGFR	Waddell, T. et al. [51]	RCT (phase 3)	553	Panitumumab + CT vs. CT alone in advanced EG cancer (REAL3 trial)	OS: 8.8 vs. 11.3 months (*p* = 0.013) PFS: 6.0 vs. 7.4 months (*p* = 0.068) ORR: 46 vs. 42% (*p* = 0.42)
	Stahl, M. et al. [52]	RCT (phase 2)	160	Panitumumab + CT vs. placebo + CT in untreated locally advanced esophagogastric cancer	Similar histological response and R0 resection rate.
	Satoh, T. et al. [53]	RCT (phase 2)	82	Nimotuzumab + irinotecan vs. irinotecan alone as second-line therapy in AGC	OS: 251 vs. 232 days (*p* = 0.978) PFS: 73 vs. 85 days (*p* = 0.567) ORR: 18.4 vs. 10.3%
	Lordick, F. et al. [54]	Phase 2	52	Cetuximab + CT as first-line therapy in metastatic GC	OS: 9.5 months PFS: 7.6 months ORR: 65%
	Moehler, M. et al. [55]	Phase 2	49	Cetuximab + irinotecan/folinic acid/5-FU as first-line therapy of HER2(+) AGC	OS: 16.5 months PFS: 9 months ORR: 46% (higher response in EGFR-expressing tumors, PTEN expression associated with longer PFS and OS)
	Lordick, F. et al. [56]	RCT (phase 3)	904	Cetuximab + capecitabine-cisplatin vs. capecitabine-cisplatin in unresectable or metastatic GC or EGJ cancer (EXPAND trial)	PFS: 4.4 vs. 5.6 months (*p* = 0.32)
	Zhang, X. et al. [57]	Phase 2	47	Cetuximab + cisplatin/capecitabine in untreated unresectable or metastatic GC	OS: 10.8 months PFS: 5.2 months ORR: 53.2%
	Liu X et al. [58]	Phase 2	61	Cetuximab + modified FOLFIRI as second-line therapy in metastatic GC	OS: 8.6 months ORR: 33.3%
VEGF	Wilke, H. et al. [59]	RCT (phase 3)	665	Ramucirumab + paclitaxel vs. placebo + paclitaxel as second-line therapy in AGC	OS: 9.6 vs. 7.4 months (*p* = 0.017) PFS: 4.4 vs. 2.9 months (*p* < 0.0001) ORR: 28 vs. 16% (*p* = 0.0001)
	Ohtsu, A. et al. [60]	RCT (phase 3)	774	Bevacizumab + CT vs. placebo + CT as first-line therapy in AGC (AVAGAST trial)	OS: 12.1 vs. 10.1 months (*p* = 0.1002) PFS: 6.7 vs. 5.3 months (*p* = 0.0037) ORR: 46 vs. 37.4% (*p* = 0.0315)
	Meulendijks, D. et al. [61]	Phase 2	60	Bevacizumab + CT as first-line therapy in HER2(−) GC	OS: 12 months PFS: 8.3 months ORR: 70%
	Meulendijks, D. et al. [62]	Phase 2	25	Bevacizumab + trastuzumab + CT as first-line therapy in HER2(+) AGC	OS: 17.9 months PFS: 10.8 months ORR: 74%
VEGF PDGF	Moehler, M. et al. [63]	Phase 2	51	Sunitinib monotherapy in pretreated AGC	OS: 5.8 months PFS: 1.3 months ORR: 4%
	Moehler, M. et al. [64]	RCT (phase 2)	91	Sunitinib + FOLFIRI vs. placebo + FOLFIRI as second- or third-line therapy in AGC	OS: 10.4 vs. 8.9 months (*p* = 0.21) PFS: 3.5 vs. 3.3 months (*p* = 0.66)
FGFR VEGF PDGF	Won, E. et al. [65]	Phase 2	32	Nintedanip as second-line therapy in metastatic EG cancer	OS: 14.2 months PFS: 1.9 months ORR: 0%
FGFR	Van Cutsem, E. et al. [66]	RCT	71	AZD4547 vs. paclitaxel as second-line therapy in AGC with FGFR2 polysomy or gene amplification (SHINE study)	OS: 5.5 vs. 6.6 months (*p* = 0.8156) PFS: 1.8 vs. 3.5 months (*p* = 0.9581) ORR: 2.6 vs. 23.3% (*p* = 0.9970)
HGFR/MET	Iveson, T. et al. [67]	RCT (phase 2)	121	Rilotumumab (2 different concentrations) vs. placebo in advanced or metastatic GC	PFS: 5.7 vs. 4.2 months (*p* = 0.016)
	Zhu, M. et al. [68]	RCT (phase 2)	121	Rilotumumab + ECX vs. placebo + ECX in MET-positive patients	High rilotumumab vs. placebo vs. low rilotumumab: OS: 13.4 vs. 5.7 and 8.1 months (*p* = 0.017) PFS: 7.0 vs. 4.4 and 5.5 months (*p* = 0.017)
	Catenacci, D.V. et al. [69]	RCT (phase 3)	609	Rilotumumab + epirubicin/cisplatin/capecitabine vs. placebo + epirubicin/cisplatin/capecitabine as first-line therapy in MET(+) AGC	OS: 8.8 vs. 10.7 months (*p* = 0.003) (study stopped early)
	Shah, M.A. et al. [70]	RCT (phase 3)	499	Onartuzumab + mFOLFOX6 vs. placebo + mFOLFOX6 in HER2-negative, MET-positive gastroesophageal adenocarcinoma	OS: 11.0 vs. 11.3 months (*p* = 0.24) PFS: 6.7 vs. 6.8 months (*p* = 0.43) ORR: 46.1 vs. 40.6% (*p* = 0.25)
Claudin 18.2	Sahin, U. et al. [71]	RCT (phase 2)	161	Zolbetuximab + CT + vs. CT alone in Claudin 18.2(+) advanced or recurrent GC (FAST trial)	Overall: PFS: 7.5 vs. 5.3 months (*p* < 0.0005) OS: 13.0 vs. 8.3 months (*p* < 0.0005) ≥70% Claudin 18.2(+): PFS: 9.0 vs. 5.7 months (*p* < 0.0005) OS: 16.5 vs. 8.9 months (*p* < 0.0005)
ATM	Bang, Y.J. et al. [72]	RCT (phase 2)	124	Olaparib + paclitaxel vs. placebo + paclitaxel in recurrent or metastatic GC	OS—overall: 13.1 vs. 8.3 months (*p* = 0.005) OS—ATM low: not reached vs. 8.2 months (*p* = 0.002) PFS: 3.91 vs. 3.55 months (*p* = 0.131) ORR: 26.4 vs. 19.1% (*p* = 0.162)
AKT	Bang, Y.J. et al. [73]	RCT (phase 2)	153	Ipatasertib + mFOLFOX6 vs. placebo + mFOLFOX6 in advanced or metastatic GC	PFS: 6.6 vs. 7.5 months (*p* = 0.56)
HDAC	Yoo, C. et al. [74]	Phase 2	45	Vorinostat + capecitabine + cisplatin as first-line therapy in AGC	OS: 12.7 months PFS: 5.9 months ORR: 42%
MMP9	Shah, M.A. et al. [75]	Phase 2	40	Andecaliximab + mFOLFOX6 in advanced GC	PFS: 7.8 months ORR: 48%
	Shah, M.A. et al. [76]	RCT (phase 3)	432	Andecaliximab + mFOLFOX vs. placebo + mFOLFOX	OS: 12.5 vs. 11.8 months (*p* = 0.56) PFS: 7.5 vs. 7.1 months (*p* = 0.10) ORR: 51 vs. 41%
PD-1/PD-L1	Muro, K. et al. [77]	Phase 1b	36	Pembrolizumab in PD-L1(+) AGC	ORR: 22%
	Fuchs, C.S. et al. [78]	Phase 2	259	Pembrolizumab in previously treated AGC (KEYNOTE-059 trial)	OS: 5.6 months (PD-L1(+)/(−): 5.8/4.9 months) PFS: 2.0 months ORR: 11.6% (PD-L1(+)/(−): 15.5/6.4%, *p* = 0.02)
	Kim, S.T. et al. [79]	Phase 2	61	Pembrolizumab in metastatic GC	ORR: 85.7% in MSI-H, 100% in EBV+
	Kawazoe, A. et al. [80]	Phase 2b	54	Pembrolizumab + S-1 + oxaliplatin in PD-L1(+) HER2-negative AGC	PFS: 9.4 months ORR: 72.2%
	Wang, F. et al. [81]	Phase 1b/2	76	1: Toripalimab (chemo-refractory) 2: Toripalimab + CT (CT-naïve) in AGC	1: OS: 4.8 months, PFS: 1.9 months, ORR: 12.1% 2. OS: not reached; PFS: 5.8 months; ORR: 66.7%;
	Kang, Y.K. et al. [82]	RCT (phase 3)	493	Nivolumab or placebo in CT-refractory AGC (ATTRACTION-2 trial)	OS: 5.26 vs. 4.14 months (*p* < 0.0001) PFS: 1.61 vs. 1.45 months (*p* < 0.0001) ORR: 11.2 vs. 0%
	Huang, J. et al. [83]	Phase 1	30	SHR-1210 in recurrent or metastatic GC refractory or intolerant to previous CT	ORR: 23.3%
	Moehler, M. et al. [84]	Phase 3	499	Avelumab vs. chemotherapy after first-line induction chemotherapy in patients with gastric or GEJ cancer	OS: 10.4 vs. 10.9 months (*p* = 0.18) OS in PD-L1(+): 16.2 vs. 17.7 months (*p* = 0.64)
PD-1/PD-L1 HER2	Janjigian, Y.Y. et al. [85]	Phase 2	37	Pembrolizumab + trastuzumab as first-line therapy in HER2(+) metastatic GC	PFS: 70% at 6 months
	Catenacci, D.V. et al. [86]	Phase 1b-2 trial	95	Pembrolizumab + margetuximab in locally advanced or metastastic HER2(+), PD-L1-unselected GE cancer	ORR: 18.48%
PD-1/PD-L1 CTLA-4	Kelly, R.J. et al. [87]	RCT (phase 2)	63	Durvalumab + tremelimumab vs. durvalumab alone vs. tremelimumab alone as scond-line therapy in CT-refractory AGC	OS: 9.2 vs. 3.4 vs. 7.7 months PFS: 1.8 vs. 1.6 vs. 1.7 months ORR: 7.4 vs. 0 vs. 8.3%
CIK-cells	Shi, L. et al. [88]	Non-randomized controlled trial	151	3 cycles of CIK-cell therapy vs. no CIK-cell therapy after curative gastrectomy and adjuvant chemotherapy for gastric adenocarcinoma	Intestinal type— 5-year OS: 46.8 vs. 31.4%, *p* = 0.045 5-year DFS: 42.4 vs. 15.7%, *p* = 0.023 Diffuse or mixed-type— 5-year OS: 7.4 vs. 7.7%, *p* = 0.97 5-year DFS: 3.7 vs. 0%, *p* = 0.96

Studies with biomarkers and relevance as to molecular targeted therapies are listed and grouped depending on the targeted biomarker. Study design, patient number, treatment aim, and treatment outcomes including overall survival, progression-free survival and overall response rate, if available, are shown. Abbreviations: AGC—advanced gastric cancer; CIK-cells—cytokine-induced killer cells; CPS—combined positivity score; CT—chemotherapy; DFS—disease-free survival; EGFR—epidermal growth factor receptor; EGJC—esophagogastric junction cancer; FGFR—fibroblast growth factor receptor; GC—gastric cancer; HDAC—histone deacetylase; HER2—human epidermal growth factor receptor 2; HGFR—hepatocyte growth factor receptor; MMP9—matrix metalloproteinase-9; ORR—overall response rate; OS—overall survival; PARP—poly ADP ribose polymerase; PD-1—programmed cell death protein 1; PD-L1—programmed cell death ligand 1; PDGF—platelet-derived growth factor; PFS—progression-free survival; RCT—randomized controlled trial; VEGF—vascular endothelial growth factor.

**Table 2:** Biomarkers and their impacts on outcomes.

Biomarker	Study	Design	Patient Number	Aim	Outcome
HER2	Iqbal, S. et al. [44]	Phase 2	47	Lapatinib as first-line therapy in advanced or metastatic GC	HER2(+) vs. HER(−): OS 6.8 vs. 3.0 months (*p* = 0.0031) IL-8 high vs. IL-8 low expression: OS 3.0 vs. 5.6 months (*p* = 0.016)
	Shitara, K. et al. [19]	Prospective observational study	364	Impact of HER2 status and trastuzumab treatment on prognosis of AGC	HER2(+) + trastuzumab vs. HER(−): OS 24.7 vs. 13.9 months (*p* = 0.03) HER2(+) w/o trastuzumab vs. HER2(−): OS 13.5 vs. 13.9 months (*p* = 0.091)
	Okines, A.F. et al. [11]	RCT	415	Prognostic and predictive impact of HER2 status (tissue samples from MAGIC trial)	HER2 status not prognostic and not predictive for response to CT
	Matsumoto, T. et al. [13]	Phase 2	89	HER2 expression in AGC with extensive LNM, correlation between HER2 status and survival	HER2(+) vs. HER2(−): 3-year OS 66.7 vs. 38.7% (*p* = 0.022) Multivariate analysis: HER2 status not prognostic
	Press, M.F. et al. [14]	RCT	487	Screening of adenocarcinoma for HER2-amplification, lapatinib in HER2(+) EG cancer	16.1% HER2 amplification, HER2 amplification levels correlated with PFS (*p* = 0.035), but not with OS
	Feizy, A. et al. [16]	Prospective observational study	210	Association of HER2 expression and survival	No association between HER2 expression and survival (*p* = 0.88)
	Kim, S.T. et al. [89]	Phase 2	32	Capecitabine + oxaliplatin + lapatinib in HER2(+) AGC	High vs. low level HER2 amplification: predictive for treatment response (*p* = 0.02)
	Shah, M.A. et al. [23]	RCT (phase 2/3)	182	Biomarker analysis of the GATSBY study: Trastuzumab emtansine vs. taxane as second-line therapy in HER2-positive AGC	High vs. low HER2 expression associated with longer OS; high HER2 expression predictor of OS
EGFR HER2	Sanchez-Vega, F. et al. [50]	Phase 2	20	Afatinib in trastuzumab-resistant HER2(+) EG cancer	Treatment response associated with EGFR + HER2 coamplification
EGFR	Luber, B. et al. [90]	Phase 2	39	Cetuximab + oxaliplatin/leucovorin/5-fluorouracil in 1st line metastatic EGJC or GC	Increased EGFR gene copy numbers associated with better OS (*p* = 0.011)
	Moehler, M. et al. [55]	Phase 2	49	Cetuximab + irinotecan/folinic acid/5-FU as first-line therapy of HER2(+) AGC	EGFR-expressing vs. nonexpressing tumors: ORR 84 vs. 23% (*p* = 0.041)
	Zhang, X. et al. [57]	Phase 2	47	Cetuximab + cisplatin/capecitabine in untreated AGC	High vs. low EGFR expression: OS: 16.6 vs. 9.5 months (*p* = 0.12), PFS: 7.1 vs. 4.0 months (*p* = 0.078)
	Liu, X. et al. [58]	Phase 2	61	Cetuximab + modified FOLFIRI in metastatic GC	EGFR(+) vs. EGFR(−): similar ORR and OS
	Stahl, M. et al. [52]	RCT (phase 2)	160	Panitumumab + CT vs. placebo + CT in untreated locally advanced EG cancer	Shorter PFS and OS with EGFR expression
VEGF	Moehler, M. et al. [63]	Phase 2	51	Sunitinib monotherapy in pretreated AGC	VEGF-C expression vs. no expression: PFS: 1.2 vs. 2.9 months (*p* = 0.012)
	Van Cutsem, E. et al. [91]	RCT	774	Bevacizumab + CT vs. placebo + CT (AVAGAST study), correlations between BM and clinical outcomes	Placebo group: baseline low vs. high VEGF-A: OS: 12.9 vs. 8.3 months. Bevacizumab group: baseline high vs. low VEGF-A: higher OS (*p* = 0.07)
	Moehler, M. et al. [64]	RCT (phase 2)	91	Sunitinib + FOLFIRI vs. placebo + FOLFIRI as second- or third-line therapy in AGC	Baseline low vs. high VEGF-A: PFS: 166 vs. 91 days (*p* = 0.017) Baseline low vs. high VEGFR2: PFS: 107 vs. 167 days (*p* = 0.044)
	Liu, X. et al. [58]	Phase 2	61	Cetuximab + modified FOLFIRI in metastatic GC	Low vs. high baseline plasma VEGF: ORR: 55 vs. 5.3% (*p* = 0.001), OS: 12 vs. 5 months (*p* < 0.0001), PFS: 14.0 vs. 6.8 months (*p* = 0.035)
	Van Cutsem, E. et al. [92]	RCT	637	Biomarker analysis from RAINBOW trial (2nd line ramucirumab + CT vs. placebo + CT in AGC)	VEGF not predictive for ramucirumab efficacy.
FGFR	Kim, S.T. et al. [93]	Phase 2	66	Pazopanib + CT in metastatic or recurrent GC	FGFR2(+) vs. FGFR2(−): PFS: 8.5 vs. 5.6 months (*p* = 0.05) OS: 13.2 vs. 11.4 months (*p* = 0.055) ORR: 85.7 vs. 59.6%
	Won, E. et al. [65]	Phase 2	32	Nintedanip as second-line therapy in metastatic EG cancer	FGFR2(+) vs. FGFR2(−): PFS: 3.5 vs. 1.9 months (*p* = 0.92)
HGFR/MET	Stahl, M. et al. [52]	RCT (phase 2)	160	Panitumumab + CT vs. placebo + CT in untreated locally advanced EG cancer	Shorter PFS and OS with MET expression.
	Sanchez-Vega, F. et al. [50]	Phase 2	20	Afatinib in trastuzumab-resistant HER2(+) EG cancer	Resistance associated with MET amplification.
PD-1/PD-L1	Fuchs, C.S. et al. [78]	Phase 2	259	Pembrolizumab in previously treated unselected AGC (KEYNOTE-059 trial)	PFS: 2.1 vs. 2.0 months in PD-L1(+) vs. PD-L1(−) ORR: 15.5 vs. 6.4% in PD-L1(+) vs. PD-L1(−)
	Kim, S.T. et al. [79]	Phase 2	61	Pembrolizumab in metastatic GC	ORR: 50 vs. 0% in PD-L1(+) vs. PD-L1(−) (*p* < 0.001)
	Wang, F. et al. [81]	Phase 1b/2	76	Toripalimab (chemo-refractory) or Toripalimab + CT (CT-naïve) in AGC	PD-L1 overexpression not associated with survival
	Huang, J. et al. [83]	Phase 1	30	SHR-1210 in recurrent or metastatic GC refractory to CT	ORR: 23.1% in PD-L1(+) and 26.7% in PD-L1(−) (*p* = 1.0)
	Choi, Y.Y. et al. [94]	RCT	592	PD-L1 expression as prognostic and predictive BM (BM study of CLASSIC trial)	Multivariate analysis of DFS: stromal PD-L1 independent prognostic factors (*p* = 0.044)

Studies with biomarkers and their impacts on outcomes are shown. Studies are grouped depending on the biomarker. Study design, patient number, treatment or study aim, and outcomes including overall survival, progression-free survival, and overall response rate, if available, are shown. Only biomarkers that have already been addressed by targeted therapies (Table 1) are shown. Abbreviations: AGC—advanced gastric cancer; CT—chemotherapy; DFS—disease-free survival; EGFR—epidermal growth factor receptor; EG—esophagogastric; EGJC—esophagogastric junction cancer; FGFR—fibroblast growth factor receptor; GC—gastric cancer; HDAC—histone deacetylase; HER2—human epidermal growth factor receptor 2; HGFR—hepatocyte growth factor receptor; LNM—lymph node metastasis; MMP9—matrix metalloproteinase-9; ORR—overall response rate; OS—overall survival; PARP—poly ADP ribose polymerase; PD-1—programmed cell death protein 1; PD-L1—programmed cell death ligand 1; PDGF—platelet-derived growth factor; PFS—progression-free survival; RCT—randomized controlled trial; TGF-a—transforming growth factor-alpha; VEGF—vascular endothelial growth factor.
